# Dietary Intakes of Traditional Foods for Dene/Métis in the Dehcho and Sahtú Regions of the Northwest Territories

**DOI:** 10.3390/nu14020378

**Published:** 2022-01-16

**Authors:** Maria Ramirez Prieto, Mylène Ratelle, Brian Douglas Laird, Kelly Skinner

**Affiliations:** School of Public Health Sciences, University of Waterloo, Waterloo, ON N2L 3G1, Canada; mj2ramirezprieto@uwaterloo.ca (M.R.P.); mratelle@uwaterloo.ca (M.R.); brian.laird@uwaterloo.ca (B.D.L.)

**Keywords:** dietary transition, traditional food, nutrition, dietary intake, sub-Arctic, Dene, Northwest Territories, Canada

## Abstract

A dietary transition away from traditional foods and toward a diet of the predominantly unhealthy market is a public health and sociocultural concern throughout Indigenous communities in Canada, including those in the sub-Arctic and remote regions of Dehcho and Sahtú of the Northwest Territories, Canada. The main aim of the present study is to describe dietary intakes for macronutrients and micronutrients in traditional and market food from the Mackenzie Valley study. We also show the trends of contributions and differences of dietary intakes over time from 1994 data collected and reported by the Centre for Indigenous People’s Nutrition and Environment (CINE) in 1996. Based on 24-h dietary recall data, the study uses descriptive statistics to describe the observed dietary intake of the Dene First Nations communities in the Dehcho and Sahtú regions of the NWT. Indigenous people in Canada, like the sub-Arctic regions of Dehcho and Sahtú of the NWT, continue to consume traditional foods, although as a small percentage of their total dietary intake. The observed dietary intake calls for action to ensure that traditional food remains a staple as it is critical for the wellbeing of Dene in the Dehcho and Sahtú regions and across the territory.

## 1. Introduction

Indigenous peoples (First Nations, Métis, and Inuit) living in Canada, including those who live in remote sub-Arctic regions, partake in subsistence harvesting—hunting, fishing, and gathering—for traditional food, and purchasing of market/commercial food with a varied emphasis [1,2,3,4]. “Traditional food” is the term more commonly used in First Nations and Métis communities, while “country food” is the preferred term of Inuit [5]. Traditional foods are considered to be any food that is locally gathered or harvested from the land and water and include sociocultural meanings and specific acquisition and processing [1,6]. Although there has been a shift in consumption of traditional foods to market foods over the years and in younger demographics, traditional food acquisition and consumption remains vital to many Indigenous people’s diets [7,8,9]. This shift away from traditional foods towards market foods is associated with high levels of saturated fat, carbohydrates, excess sugar, and sodium while being low in fiber and micronutrients [10,11,12,13], which has been described [1,14] as a form of nutrition transition [15].

Throughout Canada, a dietary transition has been observed, and while colonial and post-colonial policies, such as the Indian Act and residential schools, have disrupted Traditional Knowledge transfer and wellbeing [16], the quest for food sovereignty and self-determination is apparent in resisting this disruption [9]. Food Sovereignty is defined as the right to healthy and culturally appropriate foods and the right for people to determine how to acquire said food through agriculture, hunting, fishing, and gathering [17]. This grassroots endeavor acknowledges the colonial and post-colonial influences on food systems and strives to empower people to have the capacity to determine their food system, including the use of Traditional Knowledge and practice for the acquisition and consumption of food. Further adding to the complexity and resistance to dietary change, food security, economic status, and environmental change are interrelated with food sovereignty and consequently have influenced a shift in overall dietary profiles [1,3,4,8,14,18,19,20,21,22,23]. Likewise, the introduction of the wage system, a colonial influence, has decreased the time and energy available for traditional food acquisition [8,22]. Further, socioeconomic status is a barrier to traditional food access, with hunting being reported as too expensive by 14.7% of Yukon First Nations, 35.8% of Métis, and 42.1% of Inuit in 2006 [3], which can be attributed to costs such as boats, snowmobiles, gas, and guns [24,25]. Industrial activities contributing to anthropogenic climate change and contamination of ecosystems is also a significant factor in the shift towards market foods as climate variability is affecting migration patterns and rapid reorganizations of aquatic ecosystems [20], and traditional food has been reported to be less abundant in the Canadian Arctic [23]. Bioaccumulation of contaminants within traditional food [26,27], in conjunction with public health messaging, is associated with increased concern for contaminated traditional foods [22]. The combination of factors above, and the increase in the availability of market foods, have contributed to the observed dietary transition in many Indigenous communities within Canada. 

Likewise, the remoteness of sub-Arctic Indigenous communities within Canada has not excluded them from the dietary transition observed in other Indigenous communities nationally. While there is a variance in traditional food consumption within the Arctic and sub-Arctic regions, higher consumption of market foods relative to traditional foods has been observed for First Nations, Métis, and Inuit communities [7,10,11,14,18,19].

The Dene Nation inhabit the large area of Denendeh and includes the Dehcho and Sahtú regions of the Northwest Territories (NWT). These sub-Arctic regions are considered remote and contain rivers, lakes, and mountains, e.g., Great Bear lake, the Mackenzie Mountains, and the basin of the Mackenzie river/Dehcho river [28]. While there is literature on the observed dietary transition in sub-Arctic communities, outside of one paper that we published that focuses on game bird consumption [29], there are no exclusive data on nutritional comparisons, including comparisons over time, within and between the Dehcho and Sahtú regions of the NWT. There are also very little data on dietary intake from these regions, including recent data. As part of a larger biomonitoring project funded by the Northern Contaminants Program from the federal government, this study captures aspects of dietary intake using 24-h dietary recall data from participants collected for the Mackenzie Valley study in 2016–2018 [30,31]. Dene First Nations communities in the Dehcho and Sahtú regions of the NWT participated in the Mackenzie Valley project to study the links between nutrition, traditional foods, and contaminant exposure. The main aim of the present study is to describe dietary intakes for macronutrients and micronutrients in traditional and market food from the Mackenzie Valley study. We also show the trends of contributions and differences of dietary intakes over time from 1994 data collected and reported by the Centre for Indigenous People’s Nutrition and Environment (CINE) in 1996 in the form of an extensive community report to the Dene Nation [32] and a 2007 published paper in the Journal of Nutrition [7] to our 2016–2018 Mackenzie Valley study.

## 2. Materials and Methods

### 2.1. Recruitment

The community-based project was designed based on community consultations at the outset of the project. Discussion with communities began in 2014 and helped ensure that community needs were met and that the purpose and scope of the Mackenzie Valley Study were well understood before beginning. Researchers worked with representatives from each participating community to develop community research agreements to respect OCAP^®^ principles [33]. The agreements also detailed the expectations and responsibilities of each participating community and the research team, defined the scope of work, and informed consent procedures, data management ownership, and the expected benefits and outcomes. Methods for approaches and reporting for the project were done in collaboration with community leaders with the aim of reflecting regional and cultural attributes of the participating communities. As part of the agreements, results were returned to each community, respectively, within 12 months of data collection. Individual participants received their own results, and aggregate results were presented during community public meetings, to local leadership (e.g., community councils), and through community reports. Logistics, participant recruitment, survey implementation, and the return of results were carried out with support from hired and trained local research coordinators in each community. Samples and survey data were collected by the local research coordinators and members of the research team who traveled to the participating communities. 

**Figure 1:**
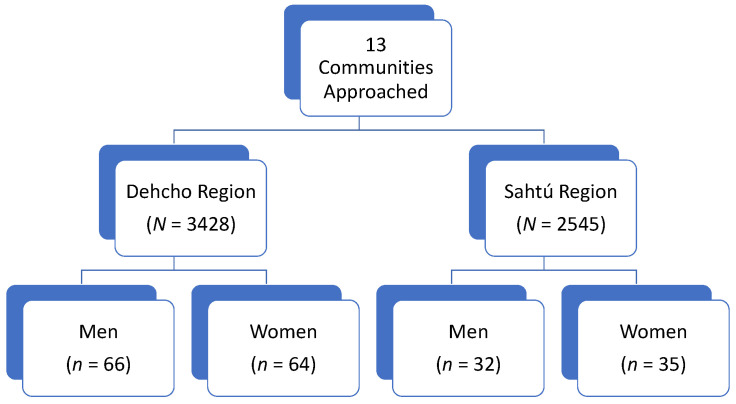
Flow chart of sampling implementation. *N* = total population of region from 2018 Summary of NWT Community Statistics [34].

Thirteen communities were approached to participate in the project. Nine accepted, with six communities in the Dehcho and three in the Sahtú regions (see Figure 1). Community members aged six years and older were eligible to participate, regardless of sex, family status, or ethnicity. Exclusion criteria for the study included community members younger than six years old, individuals who were unable to provide free and informed consent, minors who were unable to obtain the consent of their parent or guardian, and those unwilling to receive their personal results [30]. The project ran from January 2016 to March 2018, and data were collected in January 2016, November 2016, January and November 2017, and March 2018. To ensure community representation, recruitment for participants in the larger study was conducted through posters, word of mouth, and local radio interviews. In addition, sampling proportional to community size was conducted, with a minimum target of 10% of the population per community, and aimed to represent the sex and age distribution of the community population. In larger communities of more than 100 residents, randomly selected households were called by phone to invite all residents in the household to participate [30]. The number of households contacted in each larger community was based on the number of individuals in each household, the total number of landlines within the community, and the community’s size. To sample at least 10% of the residents in the community, calculations assumed that 25% of contacted households would participate. Subsequently, 40% of households were randomly selected [30]. More detailed sampling strategies and procedures can be found in previously published work about the Mackenzie Valley Study [30].

Additionally, to follow a more inclusive approach, walk-in participants to the sampling clinics were welcomed. This method of open recruitment was requested by communities during the consultation stage of the project. As a result, community members were invited to come to a biomonitoring “clinic” where the samples and survey data were collected. The locations of the clinic in each community were determined in consultation with the local leadership and were often housed centrally in community centers. Once at the clinic, the participants in the broader project were invited to complete the 24-h dietary recall.

All potential participants had the project objectives and informed consent procedures explained during individual sessions with the research team and local coordinators. After this introduction to the project, participants who chose to partake in the study were asked to sign a consent form and provide basic demographic (e.g., sex, age, etc.) and personal characteristics (e.g., height, weight, smoking status, etc.). Participants could choose one, a few, or all of the six study components that involved the collection of blood, urine, and hair samples, two dietary surveys (a 24-h dietary recall and a food frequency questionnaire [35]), and a Health Messages Survey [36,37]. For individuals under 18 years of age, the research team described the project both to the minor and to their parent/legal guardian. Following an explanation, the minor was required to confirm their understanding of the project and provide verbal consent, while the parent/guardian provided written consent by signing the consent form. Translated (North Slavey) consent documents were also available upon request, and local Indigenous language experts were hired to assist participants in interpretation as needed [30]. Importantly, the 24-h dietary recall is the focus of this paper, as these data have not yet been shared or published outside of community presentations and reports.

### 2.2. 24-h Dietary Recall

For those choosing to complete the 24-h dietary recall component of the study, participants were asked in detail what they had eaten over the previous 24 h using a web-based survey of eating behaviors [38] called the WEB-Q. The WEB-Q collects dietary intake data using a 24-h dietary recall method and has been used since 2001 to survey over 20,000 Canadian students [39,40,41,42,43,44,45], including over 500 First Nations students in Ontario and Quebec [46,47,48]. The WEB-Q was validated in grades six to eight students in southern Ontario, as well as a small group of First Nations students *(n* = 25) from northern Ontario in 2004, with good relative validity when compared to dietitian-administered interviews [38]. Wein and Freeman (1995) stated that dietary recalls are much better suited to the survey needs of Indigenous communities in comparison to dietary records [49]. Limitations of 24-h recalls include recall error, underreporting of foods, and inaccurate estimation of portion sizes. The WEB-Q has several techniques to minimize the weaknesses of this form of assessment. Prompts are built-in to remind respondents of commonly forgotten items such as beverages, spreads, and snacks between meals. Photo images of various portion sizes assist in the appropriate estimation of foods consumed. The anonymity and confidentiality of the survey may promote more honesty in reporting sensitive data. When participants begin the WEB-Q, they can select foods eaten in the previous 24 h using a few methods: typing the food name and searching from an alphabetical listing of over 900 food and beverage options, searching from typical meal-based menus, or choosing a food group from the Canada Food Guide to Eating Well, which lists the foods according to food group categories [50]. Additional categories specifying combination foods, other foods, e.g., snack foods and beverages, can also be selected. For this project, local traditional foods and locally-obtained photos of these foods, as prepared, were added to the WEB-Q to assist food selection and portion size estimation. The survey also included a question as to whether the food consumed was locally harvested. The local coordinators and the members of the research team were available to help participants complete the food surveys. Following the completion of the 24-h recall WEB-Q, participants were given an overall meal summary and could make changes to their selections if they noticed any errors. The WEB-Q was only offered on the iPads in English; however, interpreters were available at the clinics in each community if a participant required translation. 

### 2.3. Analysis

Energy and nutrient content were analyzed using the Canadian Nutrient File Database [51] and, occasionally, where data were not available, commercial product information. Food group servings were analyzed online according to Canadian Nutrient File descriptions [51]. Foods that were not classified into food groups from the 2011 version of Eating Well with Canada’s Food Guide for First Nations, Inuit, and Métis [52] were categorized as Other Foods. Other Foods generally included energy-dense, nutrient-poor foods falling into five sub-categories: mostly sugar (e.g., candies), mostly fat (e.g., mayonnaise), high-salt/high-fat foods (e.g., potato chips), high-sugar/high-fat foods (e.g., pastries), high-calorie beverages (e.g., regular soft drinks), and low-calorie beverages (e.g., diet soft drinks) based on Canadian Nutrient File definitions [51]. For this study, traditional foods were considered to be any locally gathered and harvested foods, including berries, fish (e.g., whitefish), large land-based animals (e.g., moose, caribou), game birds (e.g., duck), and small game (e.g., beaver). Additional dishes that are specific to local First Nations Peoples, but made from primarily store-bought ingredients, such as bannock (frequently consumed homemade bread that is often flat, is made with flour, and fried, baked, or cooked over a fire), were not included in the traditional food category during analysis. 

This paper reports aggregate data from the 24-h dietary recall for all nine participating communities and some disaggregated data for the Dehcho and Sahtú regions and by age and sex. Results for individual communities are not shared to respect the agreements with communities and to maintain the anonymity of participants from smaller communities. Food group consumption data were examined separately from calculated values of macronutrients, vitamins, and minerals and compared to the available data from the 90 s in the region. Descriptive statistics of frequencies were conducted in Microsoft^®^ Office Excel by gender, age (6–17, 18–50, 51+ years old), and adults vs. children (with adults being 18 years old and older and children being 6–17 years old). Additionally, R Studio was used to model linear and multivariable linear regression by gender, age (<41, ≥41), and traditional food consumption status (Yes, No) but is not reported in the present study as the findings were not statistically significant. 

The paper also reports aggregate data from a 24-h dietary recall study by the CINE team conducted in 1994 in the Yukon and the NWT [7] for the purpose of discussion. The design and implementation details are provided elsewhere [7,32]. Here, we provide the general data collection methodology for the CINE study. Individuals in nine Dene/Métis communities participated in two 24-h recall surveys in the fall and winter of 1994. Approximately 1012 Dene/Métis individuals from the Dehcho and Sahtú regions were included in the CINE project [7]. Dietary intake from the 24-h recalls in which participants were asked to remember types and quantities of food consumed were dichotomized into traditional and market food, and recall data were used to calculate mean intakes of food groups as percentages of total energy. The use of a 24-h recall method of analysis and the sample population, including Dene/Métis communities from the NWT in the 1994 CINE study, make it an excellent study to temporally compare and discuss a nutrition transition within the NWT to our current 2016–2018 Mackenzie Valley project study data. 

### 2.4. Research and Ethics Licenses

Ethics approval was obtained by the University of Waterloo Research Ethics Committee (#20173, #20950), the Stanton Territorial Health Authority for Human Research (29/12/2015), and the Aurora Research Institute (#15560, #15775, #15966, #15977, #16021). Community research agreements were established between the research team and each participating community in the Mackenzie Valley study.

## 3. Results

For the Mackenzie Valley study, a total of 199 participants completed the 24-h dietary recall, with 132 from the Dehcho and 67 from the Sahtú regions. However, two participants were removed from the dataset because their reported calorie intake was outside a normal range [53]. Both participants were women and reported 10 and 10,596 calories, respectively. Therefore, 197 participants were included (*n* = 197). Participation of women and men were similar at 50.5% and 49.7%, respectively. Ages ranged from seven to 85 years old, with a mean age of 39.9 years. 

### 3.1. Nutrient Intake of Traditional Food

Table 1 provides data on macronutrient intake according to traditional and market food consumption for the Mackenzie Valley study participants. Average total energy consumption appeared to be higher in both the Dehcho and Sahtú regions in those that consumed traditional foods in the previous 24 h. Average carbohydrate intake (% energy) appeared lower, and average protein (% energy) appeared higher in those who consumed traditional foods in the previous 24 h. However, the overall average consumption of fat (% energy) and saturated fat (g) appeared similar in those who consumed traditional food and those who did not consume traditional food in the previous 24 h. Consumption difference by age is of interest as average energy consumption from traditional food as a % of total energy was observed to be higher in adults than children in the last 24 h. Table 2 indicates average energy from traditional food by age and sex within regions. Specifically, the average % energy from traditional food was 2.3 and 4.9 times greater in adults 51 years or older compared to children six to 17 years of age in women and men, respectively. While women older than 51 appeared to have the highest reported consumption of traditional food in both regions, the average % energy from traditional foods was similar in women and men, at 5.2% and 5.1%, respectively. Overall, 35% of adults in the Dehcho and Sahtú regions reported consuming traditional food in the previous 24 h while consumption by children appeared lower at 20% (see Table 3). Of adults reporting the consumption of traditional foods, more adults reported the consumption of traditional food in the Sahtú region at 42% compared to the Dehcho region at 31%. Only one child participant (7%) in the Sahtú region reported consuming traditional food, while 28% of children in Dehcho reported consuming traditional food in the previous 24 h. However, this may be due to differences in sample size.

### 3.2. Traditional Food Consumption over Time

**Figure 2:**
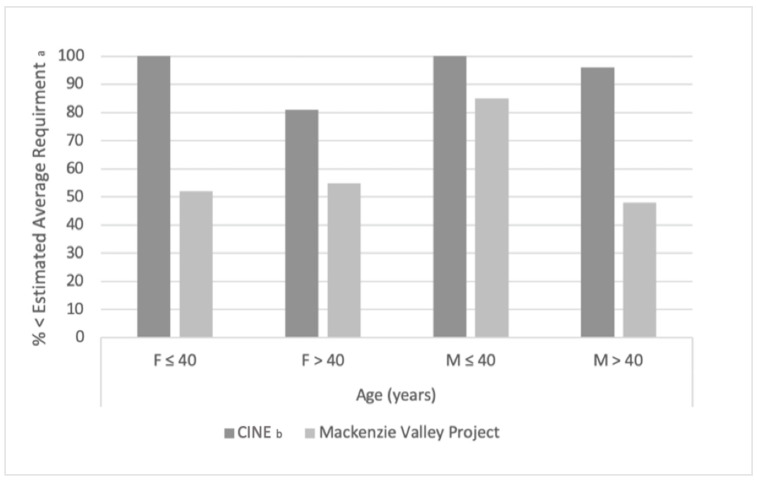
Temporal Comparison Between 1994 (CINE Study) and 2016–2018 (Mackenzie Valley Project) for Vitamin A Intake less than the Estimated Average Requirement (EAR) in Dene/Métis Adults from the NWT. ^a^ For Vitamin A, the EAR was assumed at 500 mg/day for females (F) and 625 mg/day for males (M). ^b^ From “Vitamins A, D, and E in Canadian Arctic traditional food and adult diets’’ [54]. F = female; M = male.

**Figure 3:**
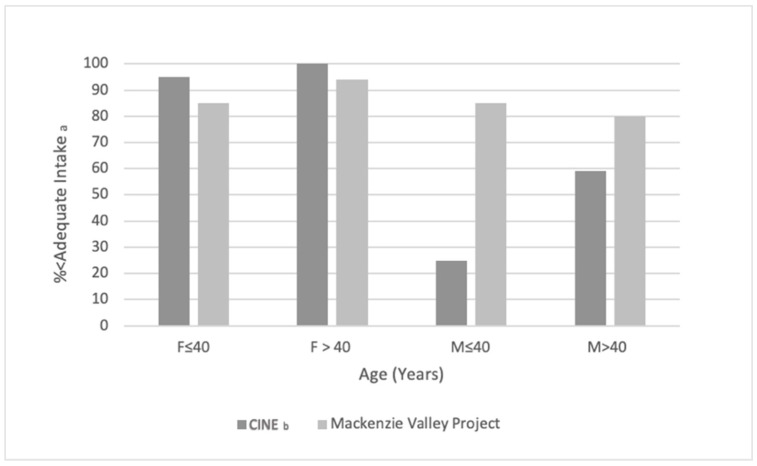
Temporal comparison between 1994 (CINE Study) and 2016–2018 (Mackenzie Valley Project) for Vitamin D intake less than the adequate intake (AI) in Dene/Métis adults from the NWT. ^a^ For Vitamin D, the AI was assumed at 5 mg/day for F and M ≤ 40 and 10 mg/day for F and M > 40. ^b^ From “Vitamins A, D, and E in Canadian Arctic traditional food and adult diets’’ [54]. F = female; M = male.

The 1994 24-h recall data reported by the CINE researchers and the current 24-h recall data from the Mackenzie Valley study provide two points in time to show observed trends in traditional food consumption over two decades apart. Further, the study depicts a subset of data from Table 1 to allow the reader to compare current dietary intake in the region compared to CINE data from 1994. Table 4 details the macronutrient and energy contribution of traditional and market foods, indicated by “with TF” and “without TF,” respectively, between the two studies. Macronutrient contributions were similar, but the current study observed a higher energy contribution of saturated fats from both traditional and market foods compared to the CINE observations. Further, Figure 2 and Figure 3 represent the % of sampled individuals who did not meet the Estimated Average Requirement (EAR) for Vitamin A or Adequate Intake (AI) of Vitamin D, and the observed low proportion of Vitamin A and D (see Figure 2 and Figure 3) intake by both men and women at all ages in the current study is of concern. The majority of participants in the present study did not obtain the EAR for Vitamin A or AI of Vitamin D. This observed low intake of Vitamin A and D, based on the proportion of participants under the EAR or AI, is also reflected in the 1994 sample. However, it appears that the % of sampled individuals who did not meet the EAR for Vitamin A in 2016–2018 is lower than the % of sampled individuals who did not meet the EAR for Vitamin A in the CINE 1994 cohort. Additionally, more women than men were meeting the EAR for Vitamin A, but conversely, the present study found men were meeting the AI of Vitamin D when sampled from 2016–2018. Of further concern is that the number of men not meeting the AI of Vitamin D appears to have increased compared to the 1994 CINE group.

## 4. Discussion

In this study, we describe the consumption of traditional and market foods from the Mackenzie Valley study and present the data alongside the consumption patterns captured by the CINE publications. To our knowledge, it is one of few studies reporting up-to-date dietary intake of traditional foods and market food for the Dehcho and Sahtú regions of the NWT. The present study’s observations display that traditional food appears to continue to be consumed in the Dehcho and Sahtú region diets, based on macronutrient intake with traditional food to be similar to those without traditional food. Likewise, the percent average energy from traditional food was similar between genders in both regions. Distinctively, women over the age of 51 years from both regions had the highest percentage of energy from traditional food. However, the Mackenzie Valley 24-h recall survey found a majority of participants reported not consuming traditional foods in the last 24 h. Moreover, the average energy of traditional foods appears to be low overall in both study regions. Importantly, there was an observed high prevalence of respondents in the current study that do not meet the EAR and AI for Vitamin A and D. The discussion will focus on studies within similar regions and First Nation groups to maximize comparability. 

In examining the results, it is evident that although the majority of energy does not come from traditional foods, it continues to be an important source of food and nutrients for the Dehcho and Sahtú region. While macronutrient breakdown was similar within the previous 24 h between those who ate traditional foods and those who did not, overall, the majority of respondents did not eat traditional foods within the last 24 h, and the average energy from traditional foods was observed to be the highest in those 51 years or older. Considering these observations, it can be suggested that a nutrition transition is occurring away from the consumption of traditional foods, as younger demographics may be demonstrating an increased reliance on market foods. Compared to the 1994 CINE study, the overall macronutrient consumption breakdown appears to be similar, while saturated fats appear to be overall higher in the present study and protein appears to be slightly lower. Like the present study, energy intakes were higher in those who consumed traditional foods in the 1994 CINE publications [7]. Further, the present Mackenzie Valley study is not alone in observing a greater consumption of market foods than traditional foods in Arctic and sub-Arctic communities. Batal and colleagues [12] found ultra-processed foods to contribute 53.9% of total energy, and only 22.7% reported consuming traditional food on the day of the 24-h recall in a sample group of 3700 First Nations in British Columbia, Alberta, Manitoba, and Ontario. In a similar study, Batal et al. sampled 92 First Nations communities across Canada and found total energy (kcal) and macronutrient and micronutrient intakes (with the exception of folate and thiamine) were significantly different between those who consumed traditional food compared to non-traditional foods within the last 24 h [55]. Importantly, total energy, protein, and micronutrients were higher in those who consumed traditional food. Distinctively, residents of the Yukon, NWT, and Nunavut were surveyed over the phone about their diet within the last seven days to investigate traditional food consumption in the Foodbook study [56]. Respondents in the NWT (60.7% of the 458) reported consuming one or more traditional foods during the previous seven days. The higher prevalence of reported traditional food consumption may be due to the seven-day period, compared to the 24-h recall survey design of other studies, including the current study. Importantly, traditional food is considered healthy according to many Indigenous communities and health professionals [53]. Accordingly, traditional food is known as fresh or minimally processed foods and is a rich source of specific nutrients. In contrast, many market foods are characterized as being highly processed and contain high amounts of fat, carbohydrate, sodium, and sugar to increase flavor [57]. Further, excess consumption of fats, carbohydrates, sodium, and sugar increases the risk of metabolic syndrome. The combination of unhealthy diets and cultural loss associated with decreased consumption of traditional food warrants concern for the observed nutrition transition and current dietary intake presented in the current study. 

Moreover, previous literature may give insights into what may have influenced the consumption of traditional foods in the current study. Phillipps and colleagues found that 54% of participants in the Sahtú region could not afford to eat a balanced meal, and 60% of participants reported that the cost of fishing and hunting prevented their household from being out on the land to harvest traditional food [58]. This may be explained through the socioeconomic barriers of hunting and fishing. Pal et al. calculated the base expenses for hunting and fishing with a final cost of 24,473 dollars (CAD) [24]. Many of these items are upfront costs, such as snowmobiles and motorboats, but also include the cost of guns, ammunition, and gas [24,58]. Hopping et al. put socioeconomic status into perspective. Participants in a household with someone employed were found to have a higher daily consumption of total traditional foods, and those on income support reported the highest odds of consuming traditional foods [10]. The NWT has a food insecurity prevalence of 21.6% [59], reflecting the high cost of living and the restrictive cost of nutritious traditional and market foods, consequently driving greater consumption of market foods. This observed consumption of more market foods than traditional foods in the present study is indicative of a nutrition transition away from a diet of mainly traditional foods and may be influenced by socioeconomic factors observed in previous studies.

Additionally, the difference in the consumption of traditional foods gives further insight into the reported nutrition transition. In the present study, those 51 years or older age had the highest consumption of country food while those aged 6–17 had the lowest consumption. Similar to the present study, Batal et al. also found differences in age and traditional food consumption, with the oldest demographics reporting the highest amount of consumed traditional food [12]. The study found 29.4% of individuals aged 51 or older reported consuming traditional food on the day of the 24-h recall. However, those aged 71 years or older reported the highest consumption of all age demographics at 35.6%. Age distribution and sample differed from the present study as they only sampled those 19 years or older and included a 71 years older group that the present study did not. However, like the present study, those 50 years or older consumed more traditional foods than their younger counterparts. Likewise, the FoodBook study conducted a seven-day recall survey and stratified by age to find those aged 0–9 had the lowest proportion of reported traditional food consumed in all territories, including the NWT [56]. Although there was no statistical significance in the consumption of traditional food by age, those 65 years or older had the highest reported consumption of traditional food within the last seven days at 71.4%. This again is similar to the trend observed in the current study and indicative of a nutrition transition away from traditional food consumed by younger demographics. 

Accordingly, investigations into different sources of food may help give insight into the differences in traditional food consumption by age. Presently, many school programs exist to combat food insecurity through breakfast programs, lunch programs, snack grants, gardening workshops, and nutrition education [60,61,62]. However, many foods mentioned to be available through food programs within the NWT consist of market foods such as yogurt, milk, fruits and vegetables, and chips with little or no mention of traditional foods [63]. While not all of the above are considered processed food [64], they take up space in the diet where traditional food could otherwise be consumed. Similar findings were observed in Indigenous youth outside of Canada as American Indigenous school children were found to consume high amounts of fat and saturated fat in both school breakfast and lunch programs [41]. Overall, school-based food programs were found to have had marginal success, and the systemic review on the diets of school-aged Indigenous youth in Canada suggested increasing traditional food to improve the dietary and cultural adequacy of school-based programs [65]. Moreover, like school food programs, community programs are primarily focused on alleviating food insecurity and are not able to prioritize the incorporation of traditional foods. Ford et al. found that traditional foods were not consistently available to Inuvik food programs and noted that the food bank was the most important source of food over the soup and bannock program [63]. 

Moreover, health risk communication and advisories are important tools for public health officials. However, improper communications, such as communication on environmental contaminants, may unnecessarily increase fear and confusion regarding traditional food. For example, Brandow found that 39% and 28% of participants in the Sahtú had heard consumption notice messaging that pregnant women and children, respectively, should avoid eating lake trout or northern pike that are larger than 60 cm [36]. Likewise, 72% of respondents indicated they had encountered messages about fish with high levels of mercury, and 31% indicated they had decreased the amount of fish they eat. Moreover, the decrease in traditional food consumption is observed through Indigenous communities, such as the Mohawk First Nation, in which fish is a cultural staple, much like in the Dehcho and Sahtú regions [35]. Hoover found that local fish consumption had dramatically decreased or ceased in the Mohawk community of Akwesasne after fish advisories were issued to reduce exposure to contaminants from nearby human industries [66]. Health messages regarding specific ages and genders may give insight into the observed lower consumption of traditional foods in younger demographics compared to those 51 years or older in the present study. 

Finally, consumption of traditional foods was found to be overall similar in men and women, with women over the age of 51 years old having the highest average percent energy from traditional foods. Importantly, men and women were represented almost equally in the sample group and age groups. Additionally, more women than men were meeting the EAR for Vitamin A, but conversely, the present study found men were meeting the AI of Vitamin D. While it appears that Vitamin A intake has improved compared to the CINE study in both men and women, men meeting the AI of Vitamin D appears to be higher than the currently observed proportion, with the percentage of men not meeting the AI appearing to be higher than that of the CINE cohort. The CINE study found women to consume more grains, sweets, fruits and vegetables, and fats compared to men, and found that men consumed slightly more meat and land animals. However, mixed dishes of traditional food and market food, sea mammals, fish, and birds contributed very similarly to the percent total energy between men and women [7]. Additionally, the food frequency questionnaire results that were also part of the larger Makenzie Valley study may help to understand the distribution of traditional food by gender. Men 19–50 years old and 51 years or older had the highest consumption of fish, land animals, birds, and large game organs, while women 51 years or older still consumed these food groups frequently and had a larger average meal size for land animals and berries compared to men their age. Young women and children had similar frequencies of consuming traditional food. Interestingly, Eeyou Istchee (Cree) women of northern Quebec had greater statistically significant odds for high traditional food intake if they were a hunter, received income security, or were older [9]. Further studies on variables influencing differences in traditional food consumption by gender are warranted. 

### 4.1. Limitations

The current study used a cross-sectional design to assess dietary intake of the Dehcho and Sahtú region using a 24-h recall survey. While a 24-h recall survey is useful in gathering data efficiently, it is difficult to generalize a dietary pattern, especially in a population in which seasons impact food availability and consumption patterns, particularly for traditional foods. The current study does not represent the seasonal variability of available traditional food. Depending on the animals available at the time (season) of the 24-h recall, it may affect the dietary intake between men and women. As such, the present study conducted surveys from January 2016 to March 2018 with the data collected in winter months, while the CINE study conducted 24-h recall surveys in March-April and October-November 1994 with the intention of capturing the lowest and highest traditional consumption periods, respectively. Subsequently, due to migration patterns of harvested animals, traditional food is often consumed more during the fall and spring hunting periods, and thus, the current study may not reflect year-round consumption patterns compared to the CINE publications. Furthermore, although the present study assesses the same regions as the CINE publications, they do not use the same cohort and, in fact, are sampled from different communities within each region. Therefore, a temporal association cannot be established between the two groups. However, the current study is the most recent to conduct dietary intake assessments of the same regions, and thus, we hope to give readers some temporal insight and updates into the dietary intake status of the Dehcho and Sahtú regions.

Further, while the WEB-Q survey was designed as an easy-to-use tool with photo-images of foods and portion sizes relative to a standard place setting, the range of food options was limited to approximately 24 animal and fish species that had contributed the most nutrients in the CINE study. In contrast, the 1992 data collection was done through one-on-one interviews and includes over 100 traditional food species specific to the region and used analyzed values of locally harvested and prepared Canadian Arctic traditional food. Finally, with both studies, the validity of a 24-h recall survey may be influenced by response bias due to a more favorable response (may be increased as the researcher is present) and the ability to recall specifically what one ate in the last 24 h [67]. 

Additionally, the design of the current study limits its ability to interpret reasons for gender and age differences, and rather we can only simply present the dietary intake data. Finally, the present study’s sample size represents about 4% to 16% of the represented communities’ populations. While the sample ratio to population is considered to be mostly low, the data collected is extremely valuable as dietary intake data in the region had not been collected for many years, and data collection in remote and rural regions is difficult.

### 4.2. Future Directions

The findings of the present study warrant a prospective cohort study investigating the nutrition transition observed in the Dehcho and Sahtú regions and other Indigenous groups within Canada to establish a temporal association between dietary intake changes and possible independent variables. Future studies should also strive to sample at multiple times of the year to accurately capture the seasonal variability of traditional food and also warrant investigation into dietary intake by gender, age, and influencing variables. 

Transformative-oriented studies with the aim of investigating promotion strategies to support the consumption of traditional foods and health communication that acknowledges and promotes the nutritional value of traditional food would be of value. Strategies meriting investigation include the usefulness of improving school food environments to foster traditional food consumption and dietary preferences of children and youth [68] and the impact of improving income and food affordability, including traditional food. Studies on assessing preferences and past experiences with health communication are warranted to understand how to develop effective communication strategies for the consumption of traditional food. 

Likewise, the present study warrants further investigation into the direct health effects of low Vitamin A and D intake and its association by gender, as the majority of the CINE and Mackenzie Valley groups did not meet the EAR or AI of Vitamin A and D, including men’s intake of Vitamin D, which appeared to decrease. 

## 5. Conclusions

The present study shows the most up-to-date dietary intake data for the Dene/Métis in the Dehcho and Sahtú Regions of the NWT and is similar to other dietary intake studies of First Nation communities within Canada. Importantly, traditional food is still being consumed, although as a small percentage of their dietary intake and less in younger demographics. Findings from the current study point to the importance of traditional food as it continues to be eaten and contributes important nutrients. The observed dietary intake calls for action to ensure that traditional food remains a staple, as it is critical for the wellbeing of Dene in the Dehcho and Sahtú regions and across the territory.

## Figures and Tables

**Table 1:** Nutrient Intake According to the Consumption of Traditional Foods (TF); Mackenzie Valley Project 2016–2018 (*n* = 197).

Region and Demographic	Energy (kcal)				Carbohydrate (% Energy)				Protein (% Energy)				Fat (% Energy)				Saturated Fat (g)				Energy from TF (% Total Energy)			
	With TF	±SD	W/O TF	±SD	With TF	±SD	W/O TF	±SD	With TF	±SD	W/O TF	±SD	With TF	±SD	W/O TF	±SD	With TF	±SD	W/O TF	±SD	With TF	±SD	W/O TF	±SD
**All Adults**	2144	913	1959	822	39	9	44	11	25	8	17	6	37	8	40	9	33	18	33	17	17	9	NA	NA
**All Children**	2190	962	1655	738	50	11	51	13	21	6	15	5	31	11	34	10	29	17	24	16	13	5	NA	NA
**Dehcho Adults**	2080	801	1872	874	40	9	44	12	26	9	16	6	36	7	41	9	29	14	33	19	18	9	NA	NA
**Dehcho Children**	2417	727	1710	883	48	8	50	14	21	6	16	5	33	9	35	11	32	15	25	17	12	6	NA	NA
**Sahtú Adults**	2235	1064	2162	656	39	10	45	10	24	5	19	4	37	8	36	9	38	21	32	14	16	8	NA	NA
**Sahtú Children**	NA	NA	1572	459	NA	NA	53	11	NA	NA	13	4	NA	NA	34	8	NA	NA	23	16	NA	NA	NA	NA

NA: not available, from 0 or only 1 participant. TF: traditional foods. W/O TF: without traditional foods.

**Table 2:** Average energy from traditional foods (% of total energy) by region and sex; Mackenzie Valley Project 2016–2018.

	All Regions			Dehcho			Sahtú		
	Total	Men	Women	Total	Men	Women	Total	Men	Women
**% Average Energy from TF**	5.1	5.1	5.2	5.0	4.5	5.5	5.4	6.3	4.5
**Age (years)**									
**6–17**	2.5	1.3	3.6	3.2	2.1	4.3	1.1	0.0	2.3
**18–50**	4.6	5.7	3.5	4.1	3.8	4.4	5.7	2.3	1.7
**51+**	7.4	6.4	8.4	7.7	7.3	8.2	6.8	5.1	8.8
**Refusal**	9.8								

TF: traditional foods. Refusal; individuals who refused to share their age were calculated within the % average energy from TF of all regions, Dehcho and Sahtú respective total, men, and women.

**Table 3:** Consumption of traditional foods (%) by region for adults and children; Mackenzie Valley Project 2016–2018, *n* = 197.

	Adults			Children		
Region	Total	% with TF	% without TF	Total	% with TF	% without TF
All (*n* = 197)	153	35	65	44	20	80
Dehcho	101	31	69	29	28	72
Sahtú	52	42	58	15	7 ^a^	93

^a^ From only one participant. TF = traditional food.

**Table 4:** Dietary intakes between 1994 (CINE Study) and 2016–2018 (Mackenzie Valley Project) for energy intake, carbohydrate, protein and fat percentage, and saturated fat in Dene/Métis adults from the NWT.

Nutrients (Average)	CINE (1994) ^a^ *n* = 1007				Mackenzie Valley Project (2016–2018) *n* = 153			
	With TF	±SD	Without TF	±SD	With TF *n* = 54	±SD	Without TF *n* = 99	±SD
**Energy (kcal)**	2261	39	2085	55	2144	913	1959	822
**Carbohydrate (% energy)**	35	1	47	1	39	9	44	11
**Protein (% energy)**	31	1	20	1	25	8	17	6
**Fat (% energy)**	34	1	39	1	37	8	40	9
**Saturated Fat (g)**	12	1	14	1	33	18	33	17

^a^ CINE Study from “Local Cultural Animal Food Contributes High Levels of Nutrients for Arctic Canadian Indigenous Adults and Children” [7]. TF = traditional food. Please note this is not a cohort group. Please see limitations within the discussion section.

## Data Availability

Restrictions apply to the availability of these data. Data was obtained from the University of Waterloo research team and are potentially available from the corresponding author with the permission of each of the participating First Nation communities, under the OCAP® principles of First Nations for the Ownership, Control, Access, and Possession of their knowledge, data, and information.
